# Matrix metalloproteinase-2 and -9 activities in the human lens epithelial cells and serum of steroid induced posterior subcapsular cataracts

**Published:** 2012-01-11

**Authors:** Bhagwat V. Alapure, Mamidipudi R. Praveen, Devarshi U. Gajjar, Abhay R. Vasavada, Trilok J. Parmar, Anshul I. Arora

**Affiliations:** Iladevi Cataract & IOL Research Center, Raghudeep Eye Clinic, Gurukul Road, Memnagar, Ahmedabad, India

## Abstract

**Purpose:**

To evaluate the level of matrix metalloproteinase (MMP)-2 and MMP-9 activities in patients with steroid induced posterior subcapsular cataract (PSC).

**Methods:**

This prospective, observational study comprised of 156 patients having either steroid induced PSC (n=50) or non-steroidal PSC (n=106) were performed to evaluate the level of MMP-2 and MMP-9 activities in the lens epithelial cells (LECs) and the serum. Anterior lens capsules harboring LECs were obtained during phacoemulsification and peripheral blood was collected from patients before administration of anesthesia. Serum was separated by centrifugation at 10,000× g for 15 min at 4 °C. The LECs and serum samples were processed to analyze MMP-2 and MMP-9 activities using succinylated gelatin assay. Quantitative real time-PCR (qRT–PCR) was performed to determine the mRNA levels of *MMP-2* and *MMP-9* in LECs. The mRNA levels were expressed as a ratio, using the delta-delta method for comparing the relative expression results between cases with steroid induced PSC and cases with non-steroidal PSC. MMP-2 and MMP-9 levels were also compared in the two groups using immunolocalization.

**Results:**

The level of MMP-2 and MMP-9 activity was found to be high in LECs and serum of cases with steroid induced PSC. Further in all steroid induced cases, a 1.4 fold increase was observed in MMP-2 activity in LECs and a 1.4 fold increase in MMP-9 activity in the serum. Both qRT–PCR and immunolocalization showed increased expression of MMP-2 and MMP-9 activity.

**Conclusions:**

MMP-2 and MMP-9 activity in both LECs and serum was significantly higher in cases with steroid induced PSC. The possible use of MMP-9 as a non-invasive biomarker in ascertaining the presence of steroid induced PSC should be evaluated using a larger sample size.

## Introduction

Posterior subcapsular cataract (PSC) is an opacity that occurs at the rear end of the lens capsule. Ultrastructural studies have suggested that the evolution of PSC is associated with fiber swelling and breakdown, which in turn is accompanied by the formation of a complex and peculiar membrane structure [1].

In 1960, Black and colleagues first noted the relationship between steroid usage and PSC [2]. It was also observed that PSC developed only after a patient had been on a high dose steroid treatment for longer than one year [3]. Moreover, the development of PSC has been observed in patients with diseases such as rheumatoid arthritis, asthma, as well as kidney transplant recipients who have continuously taken systemic steroid and immunosuppressive drug treatment. Topically applied steroids and inhaled steroids, taken for asthma, have also resulted in PSC [4]. This form of PSC as well as cortical changes due to the administration of steroidal drugs occur more often as a result of diffusion of such substances from the posterior chamber by crossing the blood-aqueous barrier, uveal inflammation, and due to disturbance of the vitreous humor [5]. The characteristic feature of steroid-induced PSC is that it shows aberrant migration of lens epithelial cells, proliferation, and suppression of differences in the lens [6].

Jobling and Augusteyn [4] suggested that administration of steroids causes an alteration in the growth factors that are reaching the lens. These alterations in the expression of growth factors might be one of the causes for steroid-induced cataract. Further, from other studies, it was understood that these growth factors are responsible for the induction of matrix metalloproteinases (MMPs) in the cells [7,8]. Reports from several other in vitro and in vivo studies using animals, human lens epithelial cells, and cell line culture have shown that transforming growth factor-beta (TGF-β) and epidermal growth factor (EGF) upregulate the expression of MMP-2 and MMP-9 [9-13].

MMPs represent a family of endopeptidases that are capable of degrading the extracellular matrix (ECM) molecules and thereby maintaining normal physiologic processes such as morphogenesis and influencing cell biologic activities [14]. Wong and colleagues [15] have shown that inhibition of MMPs prevents migration of lens epithelial cells (LECs) and contraction of the lens capsule. It echoes a similar function as that of steroids, which induce the aberrant migration of lens epithelial cells. Similarly MMPs are also found to enhance the migration of lens epithelial cells by an undefined mechanism. It was also shown that multiple MMPs are expressed in the lens and their expression is altered in several cataract phenotypes [16].

In the eye, MMPs have been examined in several ocular diseases including retinal diseases, glaucoma, corneal disorders, scleritis, uveitis, pterygium, and cataract [14,17-19]. The most widely studied MMPs in the ocular tissues are MMP-2 and MMP-9. In our previous study, we observed that MMP-9 activity was higher in cortical cataract followed by PSC and nuclear cataracts [20].

Since the role of MMPs in the genesis of PSC was not understood, in the present study, we attempted to assess the level of MMPs especially MMP-2 and MMP-9 in lens epithelial cells as well as the serum of patients with PSC.

## Methods

### Patient selection

This prospective observational study comprised 156 patients with uncomplicated age-related cataract who underwent phacoemulsification at Iladevi Cataract & IOL Research Centre Ahmedabad, India from March, 2004 to February, 2011. Informed consent was obtained from all patients. Only patients in the age group of 12–75 years with pupils dilating more than 7.0 mm and with otherwise normal eyes were included in the study. Inclusion criteria were eyes with PSC and steroid-induced PSC. Exclusion criteria were eyes with diabetes mellitus, hypertension, glaucoma, shallow anterior chambers, uveitis, high myopia (axial length <27.0 mm), pseudoexfoliation, traumatic cataract, subluxated cataract, previous ocular surgery, ocular disease, and allergy to dilating drops. The type of cataract in all the patients was recorded according to the zone of opacification. Under a slit lamp observation, the grade of the cataract was recorded based on the degree of hardness using the Emery and Little classification [21]. An experienced surgeon (A.R.V.) performed all the surgeries under topical anesthesia using a standardized technique that has been described [22] by an expert. The samples are distributed according to experimental requirement as shown in Table 1.

**Table 1:** Sample distribution of steroid-induced PSC and non-steroid induced PSC by technique.

** **	** **	**Sample**	
**Technique**	**MMPs**	**Steroid-Induced PSC**	**Non-Steroid Induced PSC**
Succinylated-gelatin assay	MMP-2 & −9	33	89
RT–PCR	MMP-2 & −9	06	06
Immunofluorescence	MMP-2	05	05
** **	MMP-9	06	06
Total	** **	50	106

### Patients medical history

A detailed medical history was collected from the 50 steroid-induced PSC patients. The mean age of the patients was 40.57±16.67 (12–68 years). To qualify for this study, the patients had be on steroid therapy for at least 2 years. Of these the following patients were on oral steroid therapy: 21 patients with renal transplantation, 8 with asthma, 2 with lung disease, 3 with arthritis, and 16 with allergy. The following steroids were administered: Wysolone (Prednisolone; Lupin Ltd, Mumbai, MH, India and Ranbaxy, Gurgaon, Haryana, India), Dexamethasone (Taj Pharmaceuticals Ltd. Mumbai, MH, India), Omnipred (Prednisolone Acetate; ALCON chemical Ltd, Bangalore, KA, India), Doxophyllin (Atman Pharmaceuticals, Mumbai, MH, India), and Cyclosporin (Taj Pharma, Mumbai, MH, India). The steroid-induced PSC patients detailed demographics shown in the Table 2. There were 106 patients with non-steroid PSC. The mean age of these patients was 53.43±9.92 (25–75 years).

**Table 2:** detailed steroid-induced PSC patients demographics.

**Disease for which steroids were prescribed**	**Number of patients**	**Steroids used**	**Use of Cyclosporin**	**Oral or topical therapy**	**Time period for which the steroid therapy lasted**	**Prescribed dosage of steroid**	**Time since PSC developed**
Renal transplantation	21	WY/CY	5 mg/day	Oral	>2–4 years	5–10 mg/day	Not recorded
Asthma	08	WY/DE/DP	-	Oral	>2–4 years	10 mg/day	Not recorded
Lung disease	02	WY/DE	-	Oral	>2 years	7–10 mg/day	Not recorded
Arthritis	03	OP/DP	-	Oral	>2–3 years	10 mg/day	Not recorded
Allergy	16	WY/DE/DP/OP	-	Oral/topical	>2–3 years	10 mg/day	Not recorded

WY- Wysolone (Prednisolone); DE- Dexamethasone; OP- Omnipred (Prednisolone Acetate); DP- Doxophyllin and CY- Cyclosporin.

### Sample processing and protein estimation

Blood was collected from patients with steroid-induced PSC (n=33) and non-steroid induced PSC (n=89) before the administration of anesthesia. From these collected blood samples, serum was separated by centrifugation at 10,000× g for 15 min at 4 °C. The serum was stored immediately at 4 °C, until used.

The curvilinear capsulorhexis, approximately 5 mm in diameter, was removed from the anterior region of the lens capsule during phacoemulsification and stored immediately at −20 °C until used. The samples were homogenized individually at 4 °C by adding 350 µl of cold 0.1 M potassium phosphate buffer (pH 7.5). All the samples were given an equal number of strokes during homogenization to obtain uniformity. The homogenate was centrifuged (Hereaus Centrifuge, Buckinghamshire, England, UK) at 10,000× g for 15 min at 4 °C, and the supernatant was stored immediately at 4 °C. The quantity of total protein in all the samples was estimated by the Lowry method [23] using BSA as a standard. Protein concentration was expressed in terms of microgram per microliter (µg/µl) of homogenate.

### Succinylation of gelatin

Gelatin was purchased from Sigma-Aldrich (St. Louis, MO) and succinylated using the procedure described previously [24]. Gelatin was dissolved in 50 mM Sodium Borate buffer with 10 mM CaCl_2_, pH 7.0 for MMP-2 and 50 mM borate buffer, pH 8.5 for MMP-9, at a concentration of 20 mg/ml. An equal amount of succinic anhydride was then gradually added to the solution and the pH of the reaction was maintained at 7.0 for MMP-2 and 8.5 for MMP-9. The succinylated gelatin was then dialyzed extensively against 50 mM sodium borate buffer, pH 7.0 and 8.5, respectively. Since dialysis resulted in dilution of the gelatin, the final concentration was determined using the Lowry’s method [23].

### Succinylated gelatin assay

Succinylated gelatin assay [25] was used for the analysis of MMP-2 and MMP-9 activities. Briefly, about 50 μl of enzyme source and 200 μg of Succinylated gelatin was added and the final volume was made up to 1.5 ml with 50 mM Sodium Borate buffer and with 10 mM CaCl_2_, pH 7.0 for MMP-2 and 50 mM borate buffer, pH 8.5 for MMP-9. A blank without substrate but with borate buffer and enzyme source was prepared for each enzyme assay. The contents were incubated at 37 °C for 30 min. About 500 μl of 0.03% solution of 2,4,6-trinitro benzene sulfonic acid (TNBSA; Sigma-Aldrich) was then added to the reaction mixture and allowed to incubate at room temperature for another 20 min. The optical density (OD) of each reaction was obtained at 450 nm using a PerkinElmer UV/VIS spectrophotometer (Molecular Devices, Menlo Park, CA). The inhibitory capacity (%) was evaluated by comparing the relative activity of the enzyme in the presence and absence of the MMP-2 inhibitor I and MMP-9 inhibitor I. Enzyme activity in the absence of the inhibitor was considered to be 100%. A standard graph was plotted using purified MMP-2 and MMP-9 (Sigma Aldrich) and was used for calculation of MMP-2 and MMP-9 activities in all the samples. The MMP-2 and MMP-9 activities were expressed in nanogram per microgram (ng/µg) of total proteins.

### Real-time PCR quantification

Total RNA was extracted from freshly collected capsulorhexis samples using TRIZOL (Invitrogen-Gibco, Grand Island, NY) according to the manufacturer’s protocol. One microgram of total RNA was reverse transcribed at 25 °C for 5 min, 42 °C for 60 min, and 70 °C for 5 min in 20 µl of 0.5 µg of oligo (dT), 20 pmol primer, 3 mM of MgCl_2_, 1 mM of dNTPs, 4 µl of Improm-II^TM^ 5× reaction buffer (Promega, Madison, WI), 1 unit of Recombinant RNasin^®^ RNase Inhibitor and 1 µl of Improm-II^™^ Reverse Transcriptase (Promega). For quantitative PCR, amplifications were performed on a Roche LightCycler 480 (Roche Ltd., Berlin, Germany) in 20 µl of reaction using 2µl of cDNA, 10µl of SYBR Green I Master, and gene specific primers at the mM concentration. The amplification was performed at 95 °C for 5 min before the first cycle, 95 °C for 5 s, and 72 °C for 30 s repeated 50 times. Quantitative PCR was performed for *MMP-2* and *MMP-9*, and glyceraldehyde-3-phosphate dehydrogenase (*GAPDH*) was used as the house-keeping gene. All the primers obtained from SABiosciences (SABiosciences, New Delhi, India) are listed in Table 3. The mRNA levels were expressed as ratios, using the delta-delta method for comparing the relative expression results between steroid-induced PSC and non-steroid induced PSC. The non-parametric Mann–Whitney U test was applied for statistical evaluation and p values <0.05 were considered statistically significant.

**Table 3:** Details of qRT–PCR primers obtained from SABiosciences.

**Number**	**Gene**	**Catalog number of primers for SA Biosciences**	**Accession number**
1	*GAPDH*	PPH00150E	NM_002046
2	*MMP-2*	PPH001510B	NM_004530
3	*MMP-9*	PPH00152E	NM_004994

### Immunostaining

Antibodies for MMP-2 and MMP-9 were obtained from Novus Biologicals (Littleton, CO) and AnaSpec (Fremont, CA), respectively. Fluorescently tagged secondary antibodies were obtained from Invitrogen. All other chemicals were purchased from Sigma-Aldrich (St. Louis, MO). Capsulorhexis specimens were placed on silane (amino-propyl-triethoxy silane)-coated glass slides with LECs facing the slide and fixed in 2% paraformaldehyde in phosphate-buffered saline (PBS) for 5 min. The samples were rinsed with PBS and chilled acetone-methanol (1:1) was used for further fixation. The capsule of the specimen was peeled with the help of forceps leaving LECs on the slides. These cells were used for immunolocalization of MMP-2 and MMP-9. Antigen retrieval of MMP-9 was performed using Proteinase k (20 µg/ml) for 1 min at 37 °C. Antigen retrieval of MMP-2 was performed using 0.25% Triton-X 100 for 10 min. Following blocking and standard washing procedures, the cells were incubated with Rabbit anti-MMP-2 (1:100) and Mouse anti-MMP-9 (1:100) antibodies for 1 h at 37 °C. The cells were then washed with PBST (PBS containing 0.05% Tween-20), and incubated with appropriate secondary antibodies tagged with Alexa fluor 546 and Alexa fluor 488 (1:200 dilution; Invitrogen, Grand Island, NY). 4', 6-diamidino-2-phenylindole (DAPI) was used to counter-stain the nuclei. An epifluorescence microscope (Axioskope II; Carl Zeiss, New York, NY) was used to observe the cells and the images were documented with a CCD camera (Cohu, San Diego, CA).

### Statistical analysis

Statistical comparisons were performed using non-parametric tests such as the Kruskal–Wallis and Mann–Whitney tests.

## Results

Of the 156 patients with uncomplicated cataract, 50 had steroid-induced PSC (mean age 40.57±16.67 [SD]) and 106 had non-steroidal PSC (mean age 53.43±9.92 [SD]).

Table 4 shows the mean value of MMP-2 and MMP-9 activities in the LECs and serum of steroid-induced PSC and non-steroidal PSC. The difference in the mean activity between steroid-induced PSC and non-steroidal PSC was statistically significant (p<0.001). Steroid-induced PSC had significantly higher activity of MMP-2 and MMP-9 in the LECs and serum as compared with non-steroidal PSC.

**Table 4:** Descriptive statistics of MMP-2 and MMP-9 activities in LECs and serum of steroid-induced PSC and non-steroid induced PSC.

** **	** **	**Mean activity (ng/µg proteins ±SD)**	
**Cataract**	**MMPs**	**LECs**	**Serum**
Steroid-induced PSC (n=33)	MMP-2	2.28±0.23	56.08±3.09
** **	MMP-9	5.96±0.20	735.97±85.35
Non-steroid PSC (n=89)	MMP-2	1.62±0.13	47.29±2.31
** **	MMP-9	5.53±0.18	540.49±14.11

MMP-2 and MMP-9 activities differ significantly between all the groups (p<0.001, ANOVA); SD, standard deviation; n- No. of Subjects, Steroid PSC- steroid-induced posterior subcapsular cataract; PSC-posterior subcapsular cataract.

Table 5 shows the results of the post-hoc statistical analysis for multiple-paired comparisons of the mean difference in MMP-2 and MMP-9 activities in LECs and serum of steroid- induced and non-steroidal PSC. A statistically significant difference was seen between steroid-induced and non-steroidal PSC (p<0.001), with the highest activity of MMP-2 and MMP-9 in the LECs and serum of steroid-induced PSC. The post-hoc analysis therefore supported the ANOVA.

**Table 5:** Multiple comparisons using the Bonferroni test for post-hoc analysis of MMP-2 and MMP-9 activities in LECs and serum of steroid-induced PSC and non-steroid induced PSC.

** **	** **	** **	**Steroid PSC (n=33)**				**PSC (n=89)**			
** **	** **	** **	**LECs**		**Serum**		**LECs**		**Serum**	
**Cataract**	**Sample**	**MMP**	**−2**	**−9**	**−2**	**−9**	**−2**	**−9**	**−2**	**−9**
Steroid PSC (n=33)	LECs	−2	** **	<0.001	<0.001	<0.001	<0.001	<0.001	<0.001	<0.001
** **	** **	−9	<0.001	** **	<0.001	<0.001	<0.001	<0.001	<0.001	<0.001
** **	Serum	−2	<0.001	<0.001	** **	<0.001	<0.001	<0.001	<0.001	<0.001
** **	** **	−9	<0.001	<0.001	<0.001	** **	<0.001	<0.001	<0.001	<0.001
Non steroid PSC (n=89)	LECs	−2	<0.001	<0.001	<0.001	<0.001	** **	<0.001	<0.001	<0.001
** **	** **	−9	<0.001	<0.001	<0.001	<0.001	<0.001	** **	<0.001	<0.001
** **	Serum	−2	<0.001	<0.001	<0.001	<0.001	<0.001	<0.001	** **	<0.001
** **	** **	−9	<0.001	<0.001	<0.001	<0.001	<0.001	<0.001	<0.001	** **

Table 6 shows the ratio of MMP-2 activity in the LECs and serum of steroid-induced and non-steroidal PSC. MMP-2 activity was increased by 0.4 and 0.18 fold in the LECs and serum of steroid-induced PSC respectively. Further, this table also shows the ratio of MMP-9 activity in the LECs and serum of steroid-induced and non-steroidal PSC. MMP-9 activity was increased by 0.4 and 0.07 fold in the LECs and serum of steroid-induced PSC respectively. Table 7 compares the levels of MMP-2 and MMP-9 activity in the LECs and serum of steroid-induced PSC. The MMP-2 ratio between the LECs and serum decreased in steroid-induced PSC (1:25) as compared to non-steroidal PSC (1:29). However, the MMP-9 ratio between the LECs and serum increased in steroid induced PSC (1:124) as compared to non-steroidal PSC (1:98).

**Table 6:** Ratio of MMP-2 and MMP-9 activities in lens epithelial cells (LECs) and serum of non-steroid and steroid-induced posterior subcapsular cataract (PSC).

** **	** **	**Mean activity (ng/µg proteins)**		** **
**Cataract**	**MMPs**	**Non-steroid PSC (n=89)**	**Steroid PSC (n=33)**	**Ratio**
LECs	MMP-2	1.62	2.28	1:1.4
** **	MMP-9	5.53	5.96	1:1.07
Serum	MMP-2	47.29	56.08	1:1.18
** **	MMP-9	540.49	735.97	1:1.36

**Table 7:** Ratio of MMP-2 and MMP-9 activities in LECs verses serum of non-steroid and steroid induced PSC.

**MMPs**	**Non-steroid PSC (LECs: Serum)**	**Steroid PSC (LECs: Serum)**	**Ratio**
MMP-2	1:29.2	1:24.5	Down
MMP-9	1:97.7	1:123.5	Up

**Figure 1:**
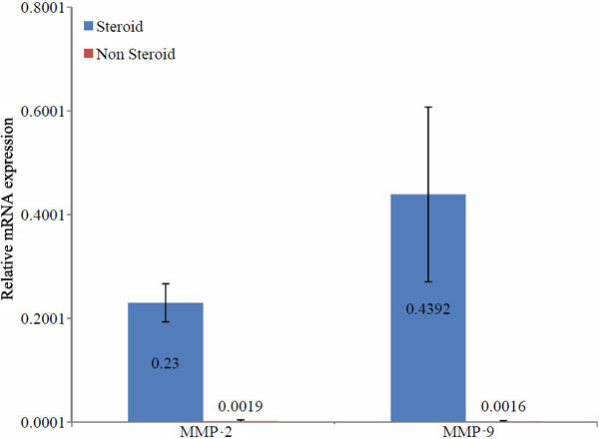
Expression of *MMP-2* and *MMP-9* mRNA in steroid and non-steroid induced PSC. The graph represents the quantitative analysis of *MMP-2* and *MMP-9* mRNA expression and both are higher in the LECs of steroid-induced PSC as compared to non-steroid induced PSC.

**Figure 2:**
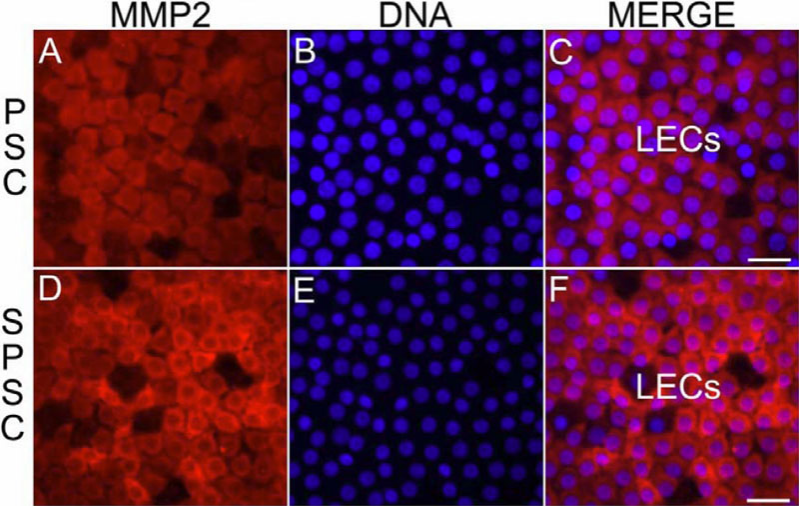
Localization of MMP-2 in lens epithelial cells (LECs) of human senile cataracts. MMP-2 levels are higher in the cytoplasm of LECs of steroid-induced PSC and MMP-2 expression was also observed in some of the cell nuclei. Non-steroid induced PSC; SPSC-steroid-induced PSC; LECs-lens epithelial cells, scale bar; 55µm.

**Figure 3:**
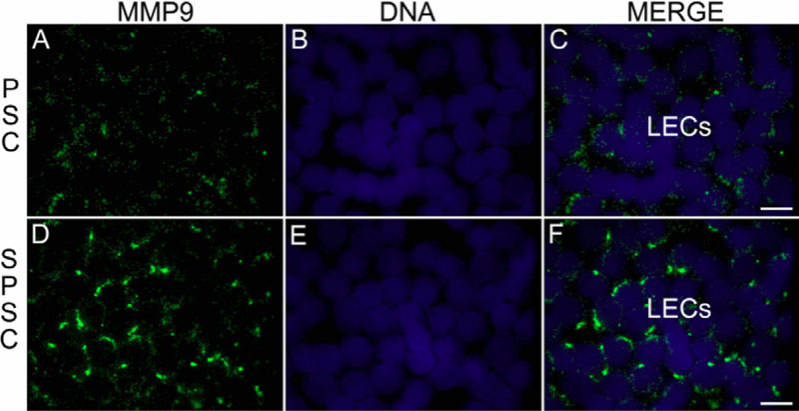
Localization of MMP-9 in lens epithelial cells (LECs) of human senile cataracts. The MMP-9 levels are higher in the cytoplasm of LECs of steroid-induced PSC. PSC-non steroid induced PSC; SPSC-steroid induced PSC; LECs-lens epithelial cells, scale bar; 35µm.

To validate the results obtained from the succinylated gelatin assay, we have used two techniques, namely qRT–PCR and immunolocation. The mRNA expression of *MMP-2* and *MMP-9* was evaluated using qRT–PCR in the LECs of steroid-induced PSC and non-steroidal PSC. The relative quantification compared to the house-keeping gene *GAPDH* showed a significant upregulation in mRNA expression of *MMP-2* and *MMP-9* in steroid-induced PSC (Figure 1) compared to non-steroidal PSC with p-values of 0.001 and 0.04, respectively. Immunolocalization of MMP-2 and MMP-9 in the LECs of steroid-induced PSC and non-steroidal PSC further validated the results of the succinylated gelatin assay. MMP-2 was observed in the nucleus and cytoplasm of the LECs (Figure 2A,D) and MMP-9 was observed in a punctuated form in the cytoplasm of the LECs (Figure 3A,D). The MMP-2 and MMP-9 levels were higher in the LECs of steroid-induced PSC (Figure 2D and Figure 3D).

## Discussion

The development of steroid-induced PSC is a complex mechanism and several attempts have been made to understand its genesis, but it has not been convincingly explained. However, in recent years, osmotic imbalance, oxidative damage, imbalance in ocular growth factors, and subsequent changes in the cellular enzymatic levels have been recognized as promising factors in the development of steroid-induced PSC [4,6]. There is growing evidence that MMPs also play an important role in many disease processes, but most of these enzymes are likely to be active at a local tissue level [26]. While other studies have reported and localized the presence of MMPs in normal as well as in cataractous lenses, the role of MMPs in the genesis of cataract has not been reported [27,28]. Several studies have suggested that growth factors [7-13] and estrogen, a steroidal hormone, are responsible for the induction of MMPs in cells [29]. A few studies have reported that oxidative stress [27] and growth factors (TGF-β) induce MMP-2 and MMP-9 activities in lens epithelial cells [11,12,30]. Oxidative stress has been shown to upregulate transcription and MMP activity in several types of cells, including hepatic stellate cells [31], human monocytes [32], and human coronary smooth muscle cells [33]. Ye and Azar [34] suggested that the penetration and migration of several cell types through the extracellular matrix (ECM) requires the activity of MMPs. Thus, it can be suggested that the abnormal induction of MMPs due to the administration of steroids or due to growth factors may result in the degradation of extracellular matrix substances that in turn help in the migration of undifferentiated anterior epithelial cells to the posterior pole forming PSC.

In our previous study, we have quantified the level of MMP-9 activity in different types of cataractous LECs, and MMP-9 activity was found to be high in cortical cataract [20]. However, no special effort was made to associate the levels of MMP-9 activity with the clinically observed steroid-induced PSC. Hence the present study was designed to measure the levels of MMP-2 and MMP-9 activities in the LECs and serum of steroid-induced PSC. We have selected serum as the second system, since we intended to analyze whether the increased level of MMP-2 and MMP-9 activities in the LECs was due to the normal physiologic status or due to the administration of drugs.

In the present study, individual anterior capsular samples (LECs) obtained by anterior capsulorhexis as well as serum of the same patients were used. As LECs are known to have low protein content, a sensitive method was required. It is well known that the levels of expression and activity of MMPs can also be determined by ELISA [35], flow cytometric analysis [36], and mRNA (mRNA) expression analysis [28]. We used succynylated gelatin assay, qRT–PCR, and immunolocalization to analyze the expression of MMP-2 and MMP-9 in steroid-induced and non-steroidal PSC. Baragi et al. [25], while evaluating the succinylated gelatin assay, found that this method relies upon the exposure of primary amines after the substrate has been degraded by gelatinases and also quantifies the specific activity. Further, the other advantages of this assay were that the protocol required only small quantities of sample and it was found to be sensitive enough to detect quantities as low as 0.1ng of MMP-2 and MMP-9. In the present study and from observations made in our previous study, we found succinylated gelatin assay a suitable and sensitive method for measuring MMP activity in LECs. The RT–PCR results in the present study showed higher expression of both MMPs in the LECs of steroid-induced PSC. Similarly we also observed higher immunohistochemical localization of MMP-2 and MMP-9 in the cytoplasm of steroid-induced LECs.

The results from the present study revealed an increase in MMP-2 and MMP-9 activities in the LECs and serum of steroid-induced PSC. The patients recruited in the present study received steroids such as Dexamethasone, Wysolone (Prednisolone), Omnipred (Prednisolone Acetate), and Doxophyllin (topical as well as oral) as well as the immunosuppressive drug (Cyclosporin). Although several studies report tantalizing results claiming that steroids influence the modulation of MMP activity, a report by Choe and coworkers [37] suggests that methylprednisolone causes emphysema-like changes in the lungs of rats due to an increase in MMP activity. Further, cyclosporine A, which is taken as an immunosuppressive drug after kidney transplantation, was also found to upregulate the expression of MMP-2 in rat hearts [38] and both MMP-2 and MMP-9 in cultured human trophoblast cells [39]. Another study by Poulalhon and colleagues [40] reported that the immunosuppressive drug, rapamycin, enhanced the expression of MMP-1. Thus, we presume that the increased activity of MMP-2 and MMP-9 in LECs may be due to the administration of such drugs.

In the case of PSC, the levels of MMP-2 activity between the LECs and serum was in the ratio of 1:29, which was shown to be reduced to 1:25 in the case of steroid-induced PSC. The reduction in MMP-2 activity in the serum of steroid-induced PSC may be attributed to the administration of steroids. However, the ratio of MMP-9 activity between the LECs and serum of steroid-induced PSC was higher than non-steroidal PSC. Since several studies have documented a rise in plasma levels of MMP-9 in cancer, hepatic and lung diseases and rheumatoid arthritis [41-44], subjects with a history of these conditions were excluded from this study. The reason for the observed increase in the level of circulating MMP-9 in the serum of steroid-induced cataract could not be understood. However, it could be attributed to the combined effect of steroids and immunosuppressive drugs. Nevertheless, the cause of increased MMP-9 activity in steroid-induced PSC remains unclear. It is possible that circulating leukocytes, macrophages [45], vascular endothelial cells [46], fibroblasts and smooth muscle cells [47,48] may contribute to the increased levels of MMP-9 activity. The absence of an increase in MMP-2 levels in the serum may reflect its more constitutive expression, whereas MMP-9 levels are more responsive to external factors such as reactive oxygen species [49], inflammatory cytokines [49], immunosuppressive drugs [38], and other steroids [37].

In summary, this is the first study that reveals an association between MMPs and steroid-induced PSC. MMP-2 and MMP-9 activities in the LECs and serum significantly differed between steroid-induced PSC and non-steroidal PSC. MMP-9 activity was higher in the serum of steroid-induced PSC; serum can be used as a secondary source to detect steroid-induced PSC. We realize that it is difficult to make wide-ranging conclusions/assumptions based on these observations in view of the small sample size used. However, this is an important starting point. Larger scale future studies will be required to clarify these findings including the link between growth factors and systemic inflammatory markers.
